# AKT inhibition overcomes rapamycin resistance by enhancing the repressive function of PRAS40 on mTORC1/4E-BP1 axis

**DOI:** 10.18632/oncotarget.3920

**Published:** 2015-04-23

**Authors:** Wenting Mi, Qing Ye, Side Liu, Qing-Bai She

**Affiliations:** ^1^ Markey Cancer Center and Department of Pharmacology and Nutritional Sciences, University of Kentucky College of Medicine, Lexington, KY, USA; ^2^ Department of Gastroenterology, Nanfang Hospital, Southern Medical University, Guangzhou, China

**Keywords:** PRAS40, 4E-BP1, mTORC1, AKT, translational regulation

## Abstract

The mTORC1 inhibitors, rapamycin and its analogs, are known to show only modest antitumor activity in clinic, but the underlying mechanisms remain largely elusive. Here, we found that activated AKT signaling is associated with rapamycin resistance in breast and colon cancers by sustained phosphorylation of the translational repressor 4E-BP1. Treatment of tumor cells with rapamycin or the AKT inhibitor MK2206 showed a limited activity in inhibiting 4E-BP1 phosphorylation, cap-dependent translation, cell growth and motility. However, treatment with both drugs resulted in profound effects *in vitro* and *in vivo*. Mechanistic investigation demonstrated that the combination treatment was required to effectively inhibit PRAS40 phosphorylation on both Ser183 and Thr246 mediated by mTORC1 and AKT respectively, and with the combined treatment, dephosphorylated PRAS40 binding to the raptor/mTOR complex was enhanced, leading to dramatic repression of mTORC1-regulated 4E-BP1 phosphorylation and translation. Knockdown of PRAS40 or 4E-BP1 expression markedly reduced the dependence of tumor cells on AKT/mTORC1 signaling for translation and survival. Together, these findings reveal a critical role of PRAS40 as an integrator of mTORC1 and AKT signaling for 4E-BP1-mediated translational regulation of tumor cell growth and motility, and highlight PRAS40 phosphorylation as a potential biomarker to evaluate the therapeutic response to mTOR/AKT inhibitors.

## INTRODUCTION

The phosphatidylinositol 3-kinase (PI3K)/AKT signaling pathway is frequently deregulated in a majority of human cancers [1]. This pathway is activated by mutations in genes that encode multiple components of the pathway or upstream activation of receptor tyrosine kinases [2]. Activation of PI3K/AKT signaling pathway has long been shown to be necessary for key features of the transformed phenotype, suggesting that inhibition of the pathway could be a useful therapeutic strategy [3]. The mammalian target of rapamycin (mTOR) protein kinase is a major component of the PI3K/AKT pathway, which controls cell growth, proliferation, survival and metabolism by integrating a variety of signals from growth factors and nutrients [4]. mTOR exerts its biological functions by the formation of two distinct protein complexes: the mTOR complex 1 (mTORC1) and the mTOR complex 2 (mTORC2). mTORC1 is composed of mTOR, raptor, mLST8, deptor and the proline-rich AKT substrate of 40 kilodaltons (PRAS40). To date, protein synthesis is the best-characterized process controlled by mTORC1 [4]. mTORC1 directly phosphorylates the eukaryotic translation initiation factor 4E (eIF4E)-binding protein 1 (4E-BP1) on Thr37 and Thr46 which act as priming sites for its subsequent phosphorylation on Ser65 and Thr70 [5]. The phosphorylation of 4E-BP1 prevents its binding to the cap-binding protein eIF4E, which allows eIF4E to participate in the assembly of the eIF4F translation initiation complex, a rate-limiting step for the cap-dependent mRNA translation and protein synthesis [6]. We and others have shown that inhibition of mTORC1 downregulates a number of certain key oncogenic mRNAs encoding proteins involved in cell-cycle progression, cell survival, angiogenesis and metastasis [7-10], and that dephosphorylated 4E-BP1 is an important biomarker for predicting a response to AKT/mTOR inhibitors [9-14].

PRAS40, first reported as substrate for AKT [15], is an inhibitory component of mTORC1 [16, 17]. Upon activation, mTORC1 can phosphorylate PRAS40 on Ser183 [18], which is in addition to the AKT-phosphorylated PRAS40 on Thr246 [15]. Phosphorylation of PRAS40 by AKT and mTORC1 results in disassociation of PRAS40 from mTORC1 and relieves its inhibitory constraint on mTORC1 activity [16-18]. Recent studies have shown that increased phosphorylation of PRAS40 is associated with malignant progression and poor prognosis in patients [19-21].

Rapamycin and its analogs (rapalogs) are allosteric inhibitors of mTORC1 via their binding to FKBP12, and were among the first mTOR-targeted therapeutics to enter the clinic [22]. However, patients whose tumors harbor a mutational activation of PI3K/AKT signaling, such as in breast, colon and prostate cancer and glioblastoma, exhibit a low response rate with rapalogs [22, 23]. It is widely believed that this inadequate therapeutic response may result from incomplete inhibition of mTORC1-mediated phosphorylation of 4E-BP1 and a concomitant activation of AKT via loss of a negative feedback mechanism [4, 22, 24, 25]. However, the molecular basis of incomplete inhibition of 4E-BP1 phosphorylation by rapamycin and how activated AKT signaling contributes to rapalogs resistance remain largely unknown.

In the present study, we demonstrate that the redundant phosphorylation of PRAS40 by both AKT and mTORC1 signaling is a novel mechanistic basis for the acquired resistance to rapamycin in cancer cells. Combined inhibition of AKT and mTORC1 is required for effective inhibition of PRAS40 phosphorylation on both Ser183 and Thr246 sites, which in turn increases the ability of PRAS40 to inhibit mTORC1-mediated 4E-BP1 phosphorylation and translation concomitant with suppression of tumor growth and cell motility. Our data uncover an important role of PRAS40 in the translational control of tumor progression and therapeutic response to mTORC1 inhibitors.

## RESULTS

### AKT inhibition profoundly enhances the inhibitory effect of rapamycin on cap-dependent translation by dephosphorylation of 4E-BP1

**Figure 1:**
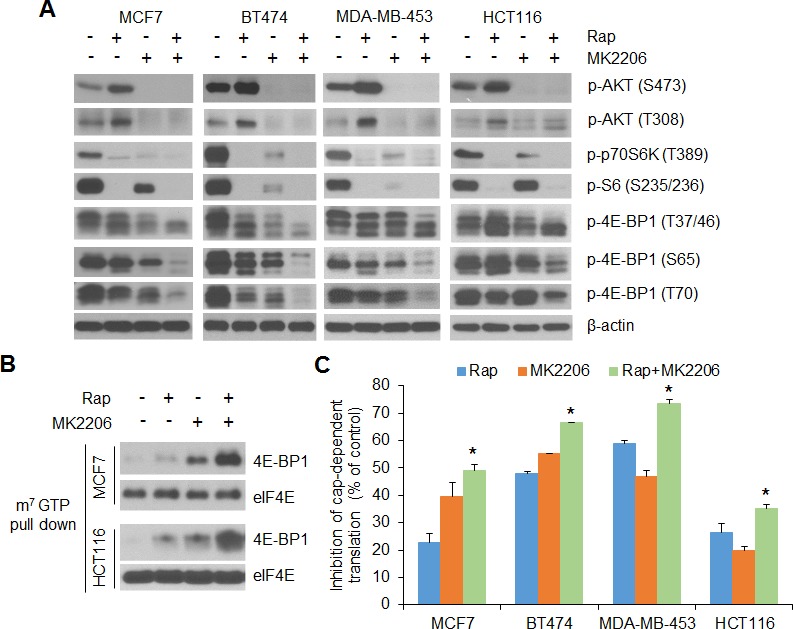
AKT inhibition profoundly enhances the inhibitory effects of rapamycin on 4E-BP1 phosphorylation and cap-dependent translation in breast and colon cancer cells **A.** The indicated cells were treated with 50 nM rapamycin (Rap) and 1 μM MK2206, alone or in combination, or DMSO as control for 12 h. Cell lysates were subjected to Western blot analysis for the indicated proteins. **B.** MCF7 and HCT116 cell lysates from (A) were precipitated with m^7^GTP sepharose beads followed by Western blot analysis for the indicated proteins. **C.** The indicated cells were transfected with a bicistronic luciferase reporter that detects cap-dependent translation of the *Renilla* luciferase gene and cap-independent poliovirus IRES-mediated translation of the firefly luciferase gene. The transfected cells were treated with the drugs as indicated in (A) for 12 h. The cap-dependent translation activity was determined as described in the Material and Methods. The results are expressed as the inhibition of cap-dependent translation relative to the DMSO-treated controls and presented as means ± S.E.M. (*n* = 3). *P < 0.05 for combination of Rap and MK2206 versus Rap or MK2206.

We and others have previously shown that the phosphorylation status of 4E-BP1 and its regulated cap-dependent translation activity are linked with cancer progression and therapeutic responses in tumors such as in breast and colon cancers with mutational activation of the PI3K/AKT signaling pathway [9-13, 26-29]. To determine whether the deregulated cap-dependent translation and feedback activation of AKT contribute to rapamycin resistance, we first examined the effects of rapamycin and the AKT inhibitor MK2206, alone and in combination, on 4E-BP1-regulated cap-dependent translation activity. In a panel of breast (MCF7, BT474, MDA-MB-453) and colon (HCT116) cancer cell lines, all with mutations in the gene that encodes the catalytic subunit of PI3K p110α (*PIK3CA*), rapamycin at 50 nM effectively inhibited phosphorylation of the mTORC1 substrate p70S6 kinase (p70S6K) and its downstream target S6 (Figure 1A). Nonetheless, rapamycin induced feedback activation of AKT, as indicated by phosphorylation on both Ser473 and Thr308 of AKT, and showed only weak inhibition on the phosphorylation of 4E-BP1 at its four mTORC1-regulated phosphorylation sites (Thr37, Thr46, Ser65, Thr70). MK2206 is a highly selective, allosteric inhibitor of AKT1, 2, and 3 that inhibits the phosphorylation of these kinases by preventing their association with the membrane [26, 30]. Treatment with MK2206 at 1 μM effectively inhibited AKT phosphorylation on both Ser473 and Thr308, but similar to rapamycin, had only a marginal effect on 4E-BP1 phosphorylation. However, MK2206 blunted the feedback activation of AKT by rapamycin and profoundly blocked phosphorylation of 4E-BP1 at all four of its phosphorylation sites in the four *PIK3CA* mutant cell lines (Figure 1A). Dephosphorylation of 4E-BP1 allows it to bind to the eIF4E-mRNA cap complex and prevents cap-dependent translation [6]. In MCF7 and HCT116 cells, treatment with either rapamycin or MK2206 slightly induced 4E-BP1 binding to the eIF4E-mRNA cap complex. However, the combination of both drugs caused marked recruitment of 4E-BP1 to the mRNA cap-complex (Figure 1B). As a result, cap-dependent translation was inhibited markedly by combination of rapamycin and MK2206 compared with either agent alone in all the four tested cancer cell lines (Figure 1C). These results suggest that in tumor cells with mutational activation of PI3K/AKT signaling pathway, combined inhibition of both AKT and mTORC1 signaling is required to effectively inhibit phosphorylation of 4E-BP1, which in turn, represses cap-dependent translation.

### 4E-BP1 mediates the effects of AKT and mTORC1 signaling on cell proliferation, survival and motility

**Figure 2:**
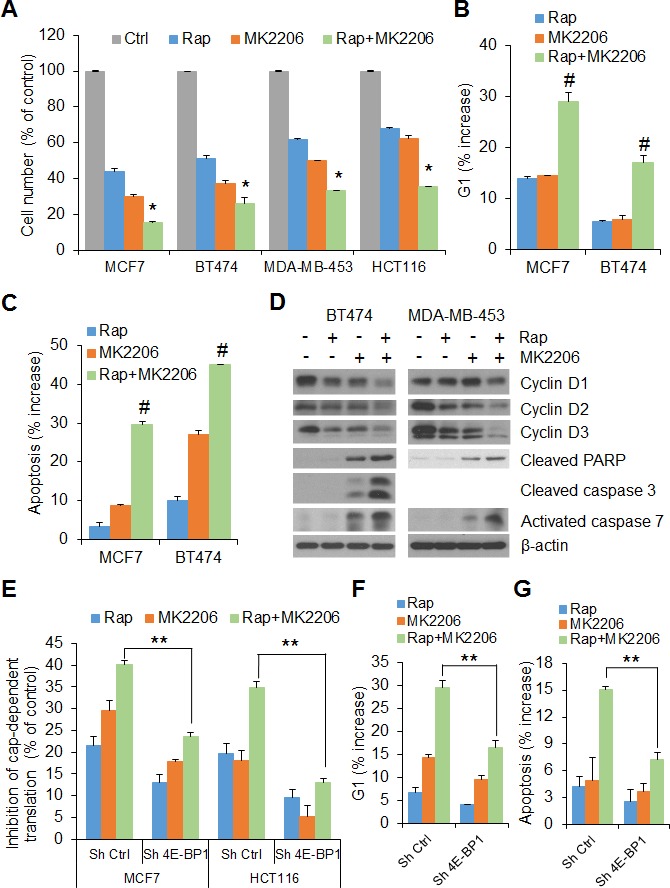
4E-BP1 integrates the effects of AKT and mTORC1 signaling on cell proliferation and survival **A.** The growth of the indicated cells was assessed after 3 days of treatment with 50 nM rapamycin (Rap) and 1 μM MK2206, alone or in combination or DMSO as control (Ctrl). The results are shown as a percentage of cell number relative to DMSO-treated control cells. *P < 0.03 for combination of Rap and MK2206 versus DMSO Ctrl, Rap or MK2206. **B, C.** Cell cycle distribution (B) and induction of apoptosis (C) in MCF7 and BT474 cells treated with 50 nM rapamycin (Rap) and 1 μM MK2206, alone or in combination or DMSO as control for 24 h (B) and 72 h (C), respectively, were analyzed by flow cytometry. The results are expressed as the increased levels of G1 (B) and apoptosis (C) by subtracting each of the DMSO-treated controls. ^#^*P* < 0.02 for combination of Rap and MK2206 versus Rap or MK2206. **D.** BT474 and MDA-MB-453 cells were treated with the drugs as indicated in Figure 1a for 12 h followed by Western blot analysis for the indicated proteins. **E.** Inhibition of cap-dependent translation activity by the indicated drugs in MCF7 and HCT116 cells with stable expression of control shRNA or 4E-BP1 shRNA was determined and analyzed as in Figure 1C. **F, G.** The increased levels of G1 (F) and apoptosis (G) by the indicated drugs in MCF7 cells with stable expression of control shRNA or 4E-BP1 shRNA were determined as in (B) and (C), respectively. **P < 0.02 for combination of Rap and MK2206 in Sh 4E-BP1 cells versus that in Sh Ctrl cells. Data shown in graphs represent the mean ± S.E.M. (*n* = 3).

To examine the functional consequences of mTORC1 and AKT cooperation on 4E-BP1-regulated translation, the effects of rapamycin and MK2206, alone and in combination, on cell proliferation were first determined. As shown in Figure 2A, simultaneous administration of MK2206 and rapamycin to MCF7, BT474, MDA-MB-453 and HCT116 cells for 72 h resulted in a marked inhibitory effect on cell proliferation compared with either agent alone. Cell cycle analysis revealed a dramatic increase of G1 phase in the MCF7 and BT474 cell lines after 24 h of treatment with the combination of AKT and mTORC1 inhibitors when compared with cells treated with either agent alone or with DMSO as control (Figure 2B and Supplementary Figure 1A). Apoptosis was assessed by staining cells with the apoptotic marker annexin V followed by FACS analysis. In BT474 and MCF7 cells, rapamycin or MK2206 alone had little or modest increase (3%-7% in MCF7 and 10%-27% in BT474) in induction of apoptosis as compared with control at 72 h after drug exposure, but the combination induced a marked induction (30% and 46% in MCF7 and BT474, respectively) of apoptosis (Figure 2C and Supplementary Figure 1B). Western blot analysis further showed that combination treatment with rapamycin and MK2206 was more effective than either agent alone in downregulating D-cyclin expression, activation of caspase-3 and/or caspase-7, key effectors of apoptosis, and increasing levels of cleaved PARP, a caspase substrate, in BT474 and MDA-MB-453 cells (Figure 2D). Collectively, these data demonstrate that AKT inhibition sensitizes tumor cells to rapamycin by enhancing G1 arrest and induction of apoptosis.

To determine whether 4E-BP1-regulated translation is directly involved in the anti-proliferative and apoptotic responses to combined inhibition of AKT and mTORC1 signaling, 4E-BP1 gene was knocked down in HCT116 and MCF7 cells (Supplementary Figure 2) using a specific shRNA target sequence as we have verified previously [9]. Combined treatment with rapamycin and MK2206 caused a 35% and 40% inhibition of cap-dependent translation in HCT116 and MCF7 control cells respectively, but had much less effect in 4E-BP1 knockdown HCT116 (12%) or MCF7 (22%) cells (Figure 2E). Furthermore, silencing 4E-BP1 expression in MCF7 and BT474 cells markedly reversed the inhibitory effects of the combination on G1 arrest and induction of apoptosis (Figure 2F, 2G and Supplementary Figures 2 and 3).

**Figure 3:**
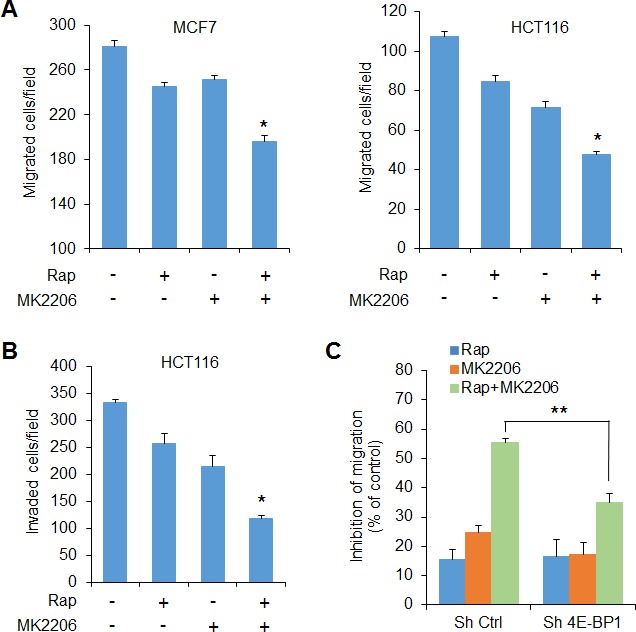
The effects of AKT and mTORC1 activation on cell migration and invasion are mediated by 4E-BP1 **A, B.** Transwell migration **A.** and invasion **B.** analyses of MCF7 and HCT116 cells were performed in the presence of 50 nM rapamycin (Rap) and 1 μM MK2206, alone or in combination, or DMSO as control for 6 h (A) and 30 h (B), respectively. The results represent the mean number of migrated (A) or invaded (B) cells per field ± S.E.M. (*n* = 3). *P < 0.03 for combination of Rap and MK2206 versus DMSO Ctrl, Rap or MK2206. **C.** Migration analysis of HCT116 cells with stable expression of control shRNA or 4E-BP1 shRNA was performed in the presence of the drugs as indicated in (A) for 6 h. The results are expressed as the inhibition of migration relative to the DMSO-treated controls. **P < 0.02 for combination of Rap and MK2206 in Sh 4E-BP1 cells versus that in Sh Ctrl cells. Data shown in graphs represent the mean ± S.E.M. (*n* = 3).

Our recent studies show that 4E-BP1-regulated cap-dependent translation also plays an important role in controlling cancer cell motility and metastasis [9, 10]. Using Boyden chamber assays described previously [9], treatment with rapamycin or MK2206 alone for 6 h had only a modest effect on MCF7 and HCT116 cell migration. However, a combination of both drugs was effective in inhibiting their migration (Figure 3A). Similar results were observed in the ability of HCT116 cells that invade through Matrigel 30 h after drug exposure (Figure 3B). Notably, knockdown of 4E-BP1 expression in HCT116 cells profoundly reduced the inhibitory effect of combined treatment on cell migration compared with that in the control cells (Figure 3C).

Taken together, these data suggest that AKT and mTORC1 signaling co-regulate 4E-BP1 phosphorylation, and that 4E-BP1 integrates the effects of AKT and mTORC1 activation on cap-dependent translation, cell proliferation, survival and motility in tumor cells with mutational activation of the PI3K/AKT signaling pathway.

### Phosphorylation of PRAS40 is a key effector of translational activation by AKT and mTORC1 signaling for cell proliferation, survival and motility

**Figure 4:**
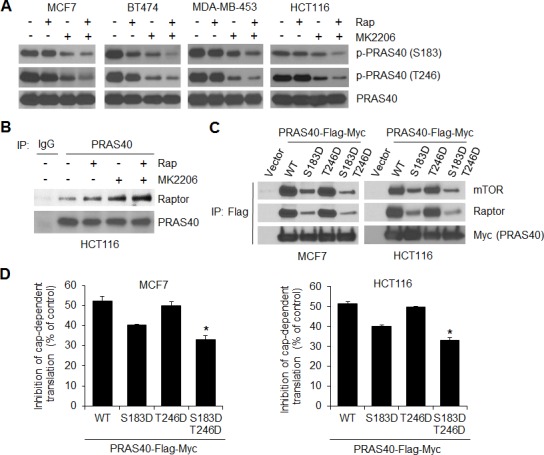
Combined inhibition of AKT and mTORC1 is required for effective inhibition of PRAS40 phosphorylation on both S183 and T246 and induction of PRAS40-repressive function on mTORC1 **A.** Cells were treated with the drugs as indicated in Figure 1A for 12 h followed by Western blot analysis for the indicated proteins. **B.** HCT116 cell lysates from **A.** were immunoprecipitated with the mouse PRAS40 antibody or control IgG followed by Western blot analysis for the indicated proteins. **C.** MCF7 and HCT116 cells were transfected with empty vector or Flag and Myc-tagged PRAS40 wild-type (WT), the PRAS40 mutants S183D and T246D, or the double mutant S183D/T246D for 48 hours. The cell lysates were immunoprecipitated with the anti-Flag antibody followed by Western blot analysis for the indicated proteins. **D.** Inhibition of cap-dependent translation activity in MCF7 and HCT116 cells transfected with the Flag and Myc-tagged PRAS40 WT, the PRAS40 mutants S183D and T246D, or the double mutant S183D/T246D, was determined and analyzed as in Figure 1C. Data shown in graphs represent the mean ± S.E.M. (*n* = 3). *P < 0.02 for PRAS40 S183D/T246D versus PRAS40 WT, PRAS40 S183D or PRAS40 T246D.

PRAS40 is a negative regulator of mTORC1 activity by competing with p70S6K and 4E-BP1 for binding to raptor [16, 18]. However, post-translational phosphorylation can inhibit the PRAS40 activity. AKT phosphorylates PRAS40 on Thr246 [15], whereas mTORC1 phosphorylates PRAS40 on Ser183 [18]. When phosphorylated, PRAS40 binding to raptor is reduced, so its ability to inhibit mTORC1 is affected as well [16, 18]. In a panel of cancer cell lines, shown in Figure 1A, inhibition of AKT with MK2206 clearly inhibited phosphorylation of PRAS40 at Thr246, whereas inhibition of mTORC1 with rapamycin did not affect the Thr246 phosphorylation (Figure 4A). However, this phosphorylation showed more inhibition with rapamycin in combination with MK2206 than with MK2206 alone. Similarly, combined treatment with rapamycin and MK2206 was required to inhibit phosphorylation of PRAS40 at Ser183 in a significant manner, whereas inhibition with either agent alone caused only modest or no effect. As such, the combination of rapamycin and MK2206 induced a greater level PRAS40 binding to raptor than did either agent alone in HCT116 cells (Figure 4B).

To establish that effective inhibition of PRAS40 activity requires both mTORC1 and AKT signaling for PRAS40 phosphorylation, we generated PRAS40 phosphorylation-mimicking mutants in which Ser183 and/or Thr246 phosphorylation sites were replaced with aspartic acids. We transfected wild-type PRAS40, the PRAS40 mutants S183D and T246D, or the double mutants S183D/T246D into MCF7 and HCT116 cells (Figure 4C, 4D). As compared to wild-type PRAS40, the S183D mutant exhibited a marked reduction in its ability to bind to raptor and mTOR, whereas the binding with the T246D mutant resembled that of the wild type. However, the greatest reduction in PRAS40 bound to the raptor/mTOR complex occurred with the double mutant (Figure 4C). In addition, the degree of PRAS40 mutant binding to the raptor/mTOR complex correlated with the extent of inhibition of cap-dependent translation (Figure 4D). The double mutant significantly attenuated the inhibitory effect on cap-dependent translation more than that induced by either S183D or T246D.

**Figure 5:**
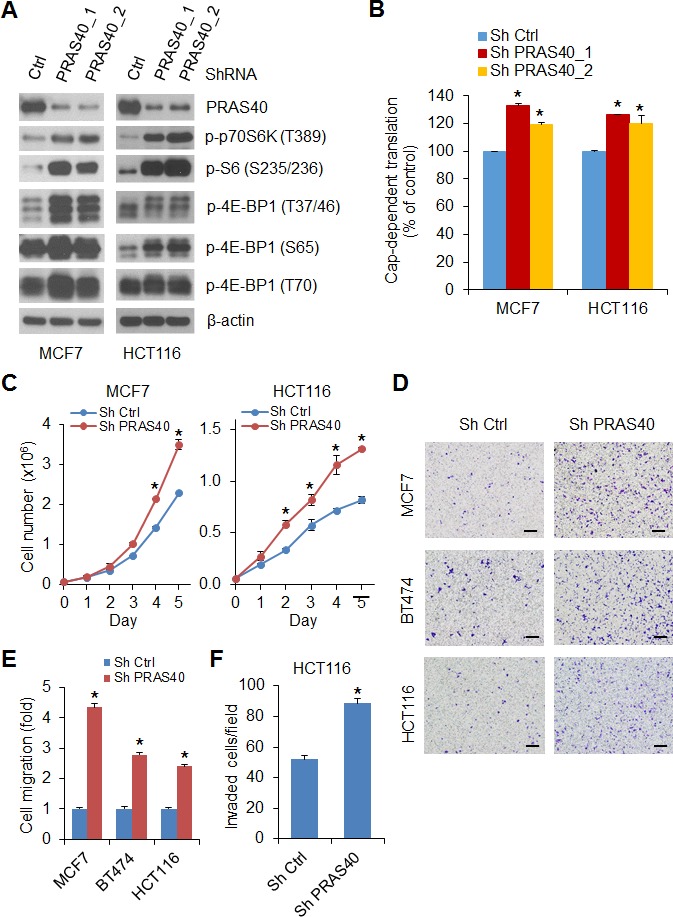
Silencing PRAS40 expression upregulates mTORC1 activity and promotes cell growth and motility **A.** Cell lysates were prepared from MCF7 and HCT116 cells with stable expression of control shRNA or two different sets of PRAS40 shRNAs, Western blot analysis was used to detect the indicated proteins. **B.** Cap-dependent translation activity in MCF7 and HCT116 cells with stable expression of control shRNA or PRAS40 shRNA was determined and analyzed as in Figure 1C. **C.** Cell proliferation analysis was performed in MCF7 and HCT116 cells with stable expression of control shRNA or PRAS40 shRNA. **D, E.** Migration analysis was performed in the indicated cell lines with stable expression of control shRNA or PRAS40 shRNA. The results are expressed as the fold change of cell migration in Sh PRAS40 cells relative to the Sh Ctrl cells. Scale bar = 500 μm. **F.** Invasion analysis of HCT116 cells with stable expression of control shRNA or PRAS40 shRNA was performed as in Figure 3B. Data shown in graphs represent the mean ± S.E.M. (*n* = 3). *P < 0.02 for Sh PRAS40 versus Sh Ctrl.

Disabling the inhibitory effects of PRAS40 by phosphorylation may exert important biologic effects in transformed cells. Knockdown of PRAS40 expression with two different sets of shRNAs in MCF7 and HCT116 cells upregulated phosphorylation of 4E-BP1, p70S6K and S6 (Figure 5A) and enhanced cap-dependent translation activity (Figure 5B), suggesting that mTORC1 was activated by PRAS40 knockdown. Furthermore, silencing PRAS40 expression in these two cell lines also promoted cell growth (Figure 5C), and exhibited a two- to five-fold increase in cell migration and invasion as compared with the control cells (Figure 5D, 5E and 5F). In addition, BT474 cells with stable knockdown of PRAS40 expression provided similar results (Figure 5D, 5E).

**Figure 6:**
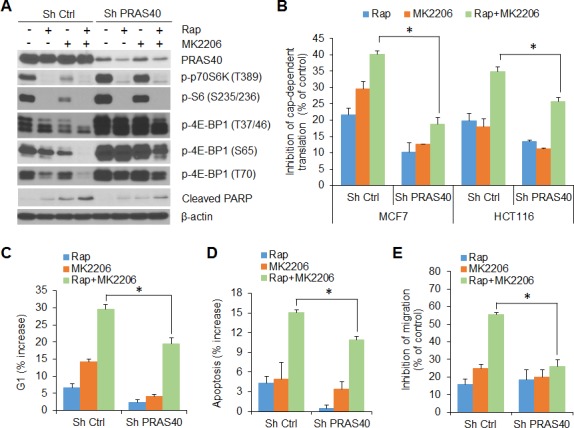
PRAS40 integrates the effects of AKT and mTORC1 signaling on 4E-BP1-regulated translation, cell proliferation, survival and motility **A.** MCF7 cells with stable expression of control shRNA or PRAS40 shRNA were treated with 50 nM rapamycin (Rap) and 1 μM MK2206, alone or in combination, or DMSO as control for 12 h. Cell lysates were subjected to Western blot analysis for the indicated proteins. **B.** Inhibition of cap-dependent translation activity by the indicated drugs in MCF7 and HCT116 cells with stable expression of control shRNA or PRAS40 shRNA, was determined and analyzed as in Figure 1C. **C, D.** The increased levels of G1 **C.** and apoptosis **D.** by the indicated drugs in MCF7 cells with stable expression of control shRNA or PRAS40 shRNA, were determined as in Figure 2B and 2C, respectively. **E.** Inhibition of migration by the indicated drugs in MCF7 cells with stable expression of control shRNA or PRAS40 shRNA, was determined and analyzed as in Figure 3C. Data shown in graphs represent the mean ± S.E.M. (*n* = 3). *P < 0.02 for combination of Rap and MK2206 in Sh PRAS40 cells versus that in Sh Ctrl cells.

Our data suggest that in cancer cells, AKT and mTORC1 cooperate to maintain phosphorylation of PRAS40, which in turn, relieves PRAS40-inhibitory constraint on mTORC1 activity; the activated mTORC1 supports cap-dependent translation by phosphorylation of 4E-BP1 and promotes cell growth and motility. To confirm this assertion, we examined the effects of AKT and mTORC1 inhibitors, alone and in combination, in control and PRAS40 knockdown MCF7 and HCT116 cells (Figure 6). Rapamycin alone effectively inhibited phosphorylation of p70S6K and S6 in both control and PRAS40 knockdown cells (Figure 6A). In contrast, treatment with the combination of rapamycin and MK2206 was required for effective inhibition of 4E-BP1 phosphorylation and cap-dependent translation in control cells (Figure 6A, 6B). However, knockdown of PRAS40 expression markedly reversed the inhibitory effects seen with the combination treatment. Similar to findings obtained with knockdown of 4E-BP1 expression (Figures 2 and 3), silencing PRAS40 expression abrogated G1 arrest, induction of apoptosis and inhibition of cell migration induced by combined treatment with rapamycin and MK2206 when compared with results obtained in control cells (Figure 6C, 6D and 6E). Collectively, these data indicate that PRAS40 integrates the effects of AKT and mTORC1 activation on 4E-BP1-mediated translational regulation of cell proliferation, survival and motility.

### AKT inhibition enhances the antitumor activity of rapamycin *in vivo*

**Figure 7:**
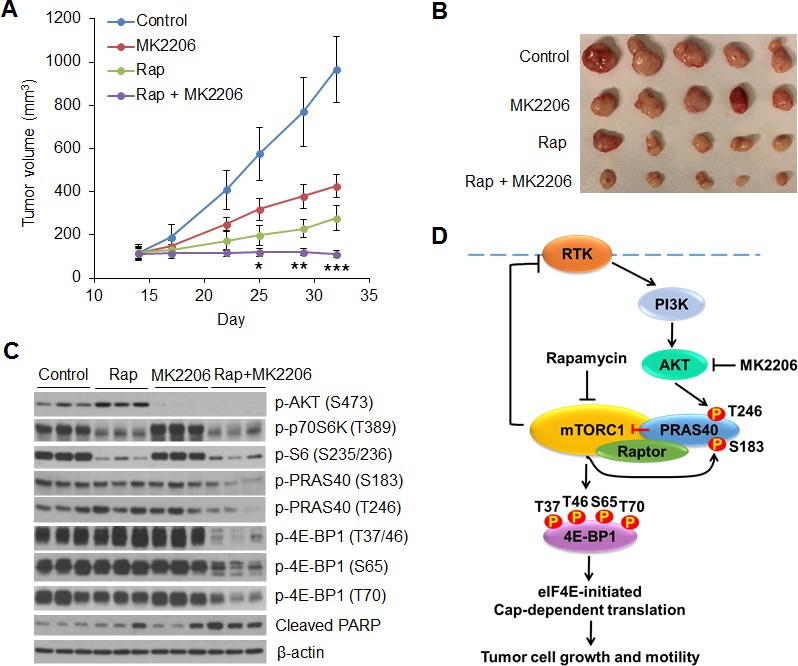
Combined inhibition of AKT and mTORC1 is required to dephosphorylate PRAS40 and 4E-BP1 and suppress tumor growth *in vivo* **A, B.** Mice bearing MCF7 xenograft tumors were treated with rapamycin (Rap) (4 mg/kg five times/week), MK2206 (100 mg/kg three times/week), combination of both drugs, or vehicle control, and tumor size was measured by caliper two times per week. The results are presented as the mean tumor volume ± S.E.M. (*n* = 5 mice/group). *P < 0.05; **P < 0.02; ***P < 0.01 for combination of Rap and MK2206 versus Rap, MK2206 or vehicle control. **C.** Representative tumors from mice in **A.** were lysed 6 h after the final treatment with the indicated drugs. Tumor lysates were subjected to Western blot analysis for the indicated proteins. **D.** A proposed model to illustrate the role of PRAS40 in integrating the effects of AKT and mTORC1 signaling on 4E-BP1-mediated translational regulation of tumor growth and therapeutic response to AKT/mTOR inhibitors. RTK, receptor tyrosine kinase.

The profound anti-proliferative and apoptotic effects of the combination of AKT and mTORC1 inhibitors *in vitro* suggest that targeting both AKT and mTORC1 signaling may be a rational strategy for the treatment of tumors with *PIK3CA* mutation. To explore the feasibility of this therapeutic strategy, we tested the safety and efficacy of inhibiting mTORC1 and AKT in *PIK3CA* mutant MCF7 tumor xenografts *in vivo*. As we and others have previously shown, the AKT inhibitor MK2206 at 100 mg/kg and the mTORC1 inhibitor rapamycin at 4 mg/kg effectively inhibit the phosphorylation of AKT and S6, respectively, in *PIK3CA* or PTEN mutant xenografts [26, 31, 32]. Nude mice bearing established MCF7 xenografts were treated with rapamycin (5 times/week at 4 mg/kg), MK2206 (3 times/week at 100 mg/kg), a combination of both drugs, or vehicle control for 3 weeks. Administration of rapamycin or MK2206 alone slowed growth of tumors, but they still grew significantly. In contrast, treatment with both drugs led to a complete suppression of tumor growth, along with modest tumor regression (Figure 7A, 7B). In addition, chronic administration of both drugs at the indicated dose and schedule was well tolerated with no weight loss in the animals (Supplementary Figure 4). Western blot analysis of tumor extracts revealed that rapamycin potently repressed phosphorylation of p70S6K and S6, but induced feedback activation of AKT (Figure 7C). MK2206 effectively inhibited AKT phosphorylation, but had no effect on the levels of phosphorylation of p70S6K and S6 (Figure 7C), which was different from what we observed *in vitro* (Figure 1A). The reason for this inconsistency is not clear but may be due to mTOR activation independent of AKT by 17β-estradiol [33] that was used for the maintenance of estrogen-dependent MCF7 xenograft tumor growth *in vivo*. Nevertheless, neither rapamycin nor MK2206 alone inhibited phosphorylation of PRAS40 and 4E-BP1 effectively, and neither drug alone induced significant PARP cleavage. In contrast, treatment with both drugs resulted in a dramatic inhibition of PRAS40 and 4E-BP1 phosphorylation and increase in PARP cleavage. These data highlight the effectiveness of concomitant inhibition of mTORC1 and AKT.

## DISCUSSION

A number of studies have shown that rapamycin and rapalogs induce feedback activation of AKT and the combination of rapamycin (or rapalogs) and AKT inhibitors results in additive or synergistic antitumor effects [34-38]. However, the molecular mechanism underlying the biological significance of the crosstalk between mTORC1 and AKT signaling during malignant transformation and therapeutic response to AKT/mTOR inhibitors has remained largely undetermined. In this study, we provide evidence to demonstrate that the convergent phosphorylation of PRAS40 (or inhibition of PRAS40 function) by activated AKT and mTORC1 signaling plays a crucial role in maintaining a transformed phenotype through 4E-BP1-regulated translation. This concept is supported by the following findings: (i) inhibition of either mTORC1 by rapamycin or AKT by MK2206 is insufficient to inhibit phosphorylation of 4E-BP1 and cap-dependent translation (Figure 1), which is associated with incomplete inhibition of PRAS40 phosphorylation on both Ser183 and Thr246 sites (Figure 4); (ii) inhibition of both mTORC1 and AKT is required for effective dephosphorylation of PRAS40 at these two sites, thereby enhancing the ability of PRAS40 to inhibit mTORC1-mediated 4E-BP1 phosphorylation and cap-dependent translation concomitant with profound suppression of cell proliferation, survival and motility as well as tumor growth; and (iii) knockdown of PRAS40 or 4E-BP1 markedly rescues these inhibitory effects induced by combined inhibition of mTORC1 and AKT. Our findings reveal a novel PRAS40/4E-BP1 axis at the crossroads of AKT and mTORC1 signaling and present a potential avenue for therapeutic control of cancer progression (Figure 7D).

The p70S6K is another well-known substrate of mTORC1. Although p70S6K and its substrate ribosomal protein S6 are also known as regulators of mRNA biogenesis and translation, a growing body of evidence, including our studies, indicate that the deregulation of cap-dependent translation downstream of mTORC1 at the level of 4E-BP1/eIF4E plays a central role in tumor formation and metastatic progression; the contribution of p70S6K1 and S6 to the oncogenic action of the mTORC1 upstream activators, AKT and/or extracellular signal-regulated kinase, appears limited [12, 27, 39, 40]. Consistent with previous studies [11, 12, 27, 39, 41], our findings support the notion that the phosphorylation status of S6 may not be a relevant biomarker to predict treatment efficacy for mTORC1 inhibitors, because loss of S6 phosphorylation by rapamycin does not always correlate with inhibition of all mTORC1 substrates such as 4E-BP1 and PRAS40.

It remains largely unknown why mTORC1 substrates show differential sensitivities to rapamycin in cancer cells. The molecular nature of rapamycin as an mTORC1 inhibitor differs from the ATP-competitive kinase inhibitors in that it forms a complex with FKBP12 and inhibits the ability of mTORC1 to phosphorylate its substrates through an unknown mechanism [22]. A recent report by Kang *et al.* suggests that the sequence composition of an mTORC1 phosphorylation site is one of the key determinants of whether the site is good or poor mTORC1 substrate within cells [42]. In this report, they found that mTORC1 could strongly phosphorylate 4E-BP1 on both Thr37 and Thr46 sites, so that even the reduced activity of rapamycin-bound mTORC1 would be sufficient to keep them phosphorylated. In other words, inhibition of mTORC1 activity by rapamycin is insufficient to inhibit 4E-BP1 phosphorylation, thereby resulting in it resistance to rapamycin. In contrast, they showed that mTORC1 weakly phosphorylates p70S6K on Thr389, which is rapamycin sensitive as the reduction of mTORC1 activity by rapamycin is sufficient to inhibit its phosphorylation. These findings suggest that maximal inhibition of mTORC1 activity is required for effective suppression of phosphorylation of rapamycin-resistant mTORC1 substrates. Interestingly, we found that inhibition of AKT activity markedly enhances the ability of rapamycin to inhibit phosphorylation of 4E-BP1 both *in vitro* and *in vivo*. Our data suggest that AKT inhibition can reverse rapamycin-resistant mTORC1 substrates being sensitive to rapamycin. Mechanistically, we found that this reversal is likely due to a more complete dephosphorylation of PRAS40, which leads to an increase in the formation of PRAS40-raptor complex by enhancing inhibition of mTORC1 activity for phosphorylation of 4E-BP1. We demonstrated that a combination of AKT and mTORC1 inhibitors is required to effectively inhibit phosphorylation of PRAS40 on both Ser183 and Thr246 sites and increases PRAS40 binding to raptor (Figure 4A, 4B). Conversely, phosphorylation of both Ser183 and Thr246 is required to reduce PRAS40 binding to raptor/mTOR complex and attenuate the inhibitory effect of PRAS40 on mTORC1/4E-BP1-mediated cap-dependent translation (Figure 4C, 4D). In addition, given that PRAS40 competes with p70S6K and 4E-BP1 for binding to raptor and inhibiting mTORC1 activity on its substrates [16, 18], the alterations in the molecular interaction among p70S6K, 4E-BP1, PRAS40, raptor, mTOR and rapamycin may yield scenarios where the ability of the substrate binding to raptor/mTOR and the sensitivity to rapamycin-induced disassociation may differ. Whether the increase of PRAS40 bound to raptor by the combination of rapamycin and AKT inhibition is required for the dissociation of 4E-BP1 rather than p70S6K from raptor remains to be determined.

It is important to note that prolonged treatment with rapamycin has been shown to inhibit AKT via disruption of mTORC2 assembly in certain cell lines [43], but in many other cell types including preclinical and clinical specimens in our present and prior studies as well as others reports, chronic administration of rapamycin could induce substantial activation of AKT signaling [22, 24, 25, 34-38, 43, 44]. In addition to the *PIK3CA* mutant tumor cells as we tested in this study, we and others also observed that in *KRAS* mutant and wild-type colon and lung cancer cells, rapamycin could also elicit AKT activation and increase the level of AKT-mediated phosphorylation of PRAS40 on Thr246 (Supplementary Figure 5) [38]. Moreover, combined inhibition of AKT and mTORC1 is also required to effectively inhibit phosphorylation of both PRAS40 and 4E-BP1 and cell growth in these cells (Supplementary Figure 5) [38]. These data suggest that co-targeting AKT and mTORC1 signaling may be a useful therapeutic strategy for many malignancies. However, a detailed analysis of different tumors with specific genotypes is needed to determine the role of PRAS40/mTORC1/4E-BP1 axis in the effectiveness and therapeutic index of this approach.

The implications of our findings suggest that the ATP-competitive mTOR kinase inhibitors (mTORKIs) that inhibit both mTORC1 and mTORC2 may provide better therapeutic outcomes when compared with rapamycin in the clinic. mTORC2 directly phosphorylates AKT at its hydrophobic motif (Ser473), which enhances the catalytic activity of AKT already phosphorylated on Thr308 [45]. In contrast to rapamycin-induced feedback activation of AKT with limited inhibitory activity on 4E-BP1 phosphorylation, the mTORKIs can effectively inhibit AKT phosphorylation on Ser473 and show much greater repression on 4E-BP1 phosphorylation [46, 47]. However, a recent report by Rodrik-Outmezguine *et al.* showed that phosphorylation of AKT at the Thr308 site and of the AKT substrates including PRAS40, GSK-3β and FOXO1/3 are only transiently repressed by mTORKIs in *PIK3CA* mutant (MCF7, BT474) and PTEN-deficient (MDA-MB-468) breast cancer cell lines [48]. Moreover, the mTORC1-mediated phosphorylation of 4E-BP1 on Thr37/Thr46 also rebounded between 8 and 24 h after treatment with mTORKIs such as AZD8055 and PP242. They found that inhibition of mTOR kinase relieves feedback inhibition of receptor tyrosine kinases, which causes subsequent activation of PI3K and re-phosphorylation of AKT on Thr308 sufficient to reactivate AKT activity and signaling, whereas combination of mTORKIs and AKT inhibitors demonstrates a more stable inhibition of 4E-BP1 phosphorylation than mTORKIs alone and dramatically induces apoptosis [48]. Consistent with these findings, our recent study also showed that although knockdown of raptor expression markedly inhibits 4E-BP1 phosphorylation, inhibition of AKT activity could further attenuate 4E-BP1 phosphorylation associated with significant antitumor effects in raptor-knockdown colon cancer cells [9]. Furthermore, additional experimental evidence has highlighted that incomplete inhibition of 4E-BP1 phosphorylation is a mechanism of primary resistance to mTORKIs [13, 49]. These studies identified that *KRAS*-mutant SW620 colon cancer cells are particularly resistant to PP242, and PP242 cannot effectively inhibit 4E-BP1 phosphorylation. Intriguingly, we found that AKT inhibition by MK2206 in combination with PP242 could elicit more profound inhibition of 4E-BP1 phosphorylation compared to the responses to PP242 alone (Supplementary Figure 6). Taken together, these data suggest that AKT kinase may be involved in the regulation of 4E-BP1 phosphorylation and translation in mTORC1-depedent and -independent manners, and that combined inhibition of mTOR and PI3K/AKT may be a promising therapeutic strategy for many malignancies. Indeed, dual inhibition of PI3K and mTORC1/2 signaling by NVP-BEZ235 or rapalogs in combination with PI3K or AKT inhibitors has demonstrated profound efficacy in a variety of preclinical models of cancers [34-37, 50-56].

In summary, our study provides new insight into the biologic and therapeutic relevance of PRAS40 in translational regulation of tumor cell proliferation, survival and motility. Our findings reveal that regulation of PRAS40 activity through cooperative AKT and mTORC1 phosphorylation of both Ser183 and Thr246 is a key process to alter the mTORC1 substrate (e.g. 4E-BP1) specificity. In addition to the phosphorylation of 4E-BP1, PRAS40 phosphorylation may also serve as a surrogate marker to evaluate the response to the PI3K/AKT/mTOR pathway inhibitors in clinic. These findings could have high translational significance and enhance our understanding of the involvement of PRAS40 and 4E-BP1 in the regulation of cancer progression and therapeutics.

## MATERIALS AND METHODS

### Cell culture, inhibitors, constructs, and lentiviral shRNA silencing

Human breast and colon cancer cell lines were obtained from the American Type Culture Collection (ATCC, Manassas, VA) and maintained in the appropriate medium with supplements as suggested by ATCC. Rapamycin and MK2206 were obtained from LC Laboratories (Woburn, MA) and Selleck (Houston, TX), respectively. The PRK5-PRAS40 construct was from Addgene (Cambridge, MA). The PRAS40 cDNA was amplified by PCR using PRK5-PRAS40 as a template and the product subcloned into the Sgf I and Mlu I sites of pCMV6-Myc-Flag (Origene, Rockville, MD). The insert was mutated using a QuikChange XLII mutagenesis kit (Stratagene, Santa Clara, CA). Mutants with Asp at Ser183 (S183D), Thr246 (T246D) and both Ser183 and Thr246 (S183D/T246D) were generated in pCMV6-Myc-Flag. All constructs were verified by DNA sequencing. Lentiviral shRNAs to human 4E-BP1 were from Open Biosystems (Lafayette, CO) and the specificity of the targeting sequences has been verified in our previous study [9]. Lentiviral shRNAs to human PRAS40 and the Non-Target Control shRNA (SHC002) were from Sigma (St Louis, MO). The accession numbers of PRAS40 ShRNA_1 and PRAS40 ShRNA_2 are TRCN0000158835 and TRCN00001666394, respectively. For establishing stable transfectants with knockdown of specific protein expression, cell lines were lentivirally infected with the indicated shRNA construct followed by selection with puromycin (2 μg/ml) for one week as described previously [9].

### Cell proliferation assay

Cell proliferation was assessed by counting the number of viable cells in response to the treatment with the indicated drugs as described previously [9].

### Cell-cycle analysis and apoptosis assay

Cells were plated in 100-mm dishes, grown overnight, and treated as indicated in figure legends. Both adherent and floating cells were harvested. For cell-cycle analysis, cell nuclei were prepared by the method of Nusse [57] and stained with ethidium bromide as described [26]. Cell cycle distribution was determined by flow cytometry. For apoptosis, cells were analyzed by flow cytometry using the Annexin V-FITC Apoptosis Detection Kit according to the manufacturer's protocol (BD Biosciences, San Jose, CA, USA).

### Migration and invasion assays

Migration and invasion assays were performed in Boyden chambers with coated collagen or Matrigel, respectively, as instructed by the manufacturer (BD Biosciences) and described previously [9].

### Western blot analysis

Cells were lysed in NP-40 lysis buffer, and analyzed by Western blot using equal total protein loading as described previously [12]. Phosphorylation-specific antibodies and antibodies for PRAS40, 4E-BP1, eIF4E, raptor, Myc tag, cleaved PARP, cleaved caspase-3 and caspase-7 were from Cell Signaling Technology (Danvers, MA). mTOR antibody (H-266) was from Santa Cruz Biotechnology (Dallas, TX) and β-actin antibody was from Sigma.

### Immunoprecipitation

PRAS40 complexes were immunoprecipitated with PRAS40 antibody (Clone 73P21, EMD Millipore, Billerica, MA) or anti-Flag antibody (Sigma) according to the procedure described by Sancak *et al* [16]. At the end, the immunoprecipitates from 0.5 to 1 mg protein of cell lysates captured with protein G Sepharose were analyzed by Western blot.

### Cap-binding assay

Cell lysates as prepared above were incubated with m^7^GTP Sepharose beads (GE Healthcare Bio-Sciences, Pittsburgh, PA) to capture eIF4E and its binding partners. Precipitates were washed three times with lysis buffer and resuspended in 2× Laemmli sample buffer followed by Western blot analysis.

### Quantification of cap-dependent translation activity

Cells (80,000) were transfected with a bicistronic luciferase reporter plasmid (0.2 μg), pcDNA3-rLuc-PolioIRES-fLuc, which directs cap-dependent translation of the *Renilla* luciferase gene and cap-independent Polio IRES-mediated translation of the firefly luciferase gene [58], in 12-well plates using X-tremeGENE Transfection Reagent (Roche Applied Science, Indianapolis, IN). After 24 h transfection, cells were treated with kinase inhibitors for the indicated times, and cell lysates were assayed for renilla luciferase and firefly luciferase activities as described [9, 12]. Cap-dependent renilla activity was normalized against cap-independent firefly activity as the internal control. The renilla/firefly luciferase luminescence ratio was calculated for cap-dependent translational activity.

### Animal studies

Female athymic nude mice (5-6 weeks old) were purchased from Taconic (Hudson, NY). Experiments were carried out under a protocol approved by the University of Kentucky Institutional Animal Care and Use Committee. MCF7 xenograft tumors were established by subcutaneously implanting 0.72 mg sustained release 17β-estradiol pellets into one flank at least 3 days before injecting 5 × 10^6^ cells suspended 1:1 (volume) with growth factor-reduced Matrigel (BD Biosciences) on the opposite side. For efficacy studies, mice were randomized among control and treated groups (*n* = 5 per group) when tumors were well-established (~150-180 mm^3^). Rapamycin was prepared in absolute ethanol at 10 mg/ml and diluted in 5% Tween-80 and 5% PEG-400 before injection. Rapamycin was administered by intraperitoneal injection at 4 mg/kg once per day, Mon-Fri as previously reported [32]. MK2206 was formulated in 30% captisol and given orally at 100 mg/kg once per day, Mon-Wed-Fri based on previous reports [31]. Control mice received a vehicle solution. Tumor dimensions were measured using a caliper and tumor volumes were calculated as mm^3^ = π/6 x larger diameter x (smaller diameter)^2^. Tumors were excised and snap frozen in liquid nitrogen, homogenized in 2% SDS lysis buffer and then processed for Western blot analysis as described previously [9, 12].

### Statistical analysis

All experiments were performed at least twice. Results are expressed as mean ± S.E.M. where applicable. A two-tailed Student's *t*-test was used to compare the intergroup. Differences between groups were considered statistically significant at *P* < 0.05.

## SUPPLEMENTARY MATERIAL FIGURES


