# Supplementary material for: AKT inhibition overcomes rapamycin resistance by enhancing the repressive function of PRAS40 on mTORC1/4E-BP1 axis
Source: Oncotarget. 2015 Apr 23;6(16):13962–77. doi: 10.18632/oncotarget.3920 (PMC4546444; doi:10.18632/oncotarget.3920)
Supplement: Supplementary file 1 [file oncotarget-06-13962-s001.pdf]

AKT inhibition overcomes rapamycin resistance by enhancing the repressive function of PRAS40 on mTORC1/4E-BP1 axis

Supplementary Material

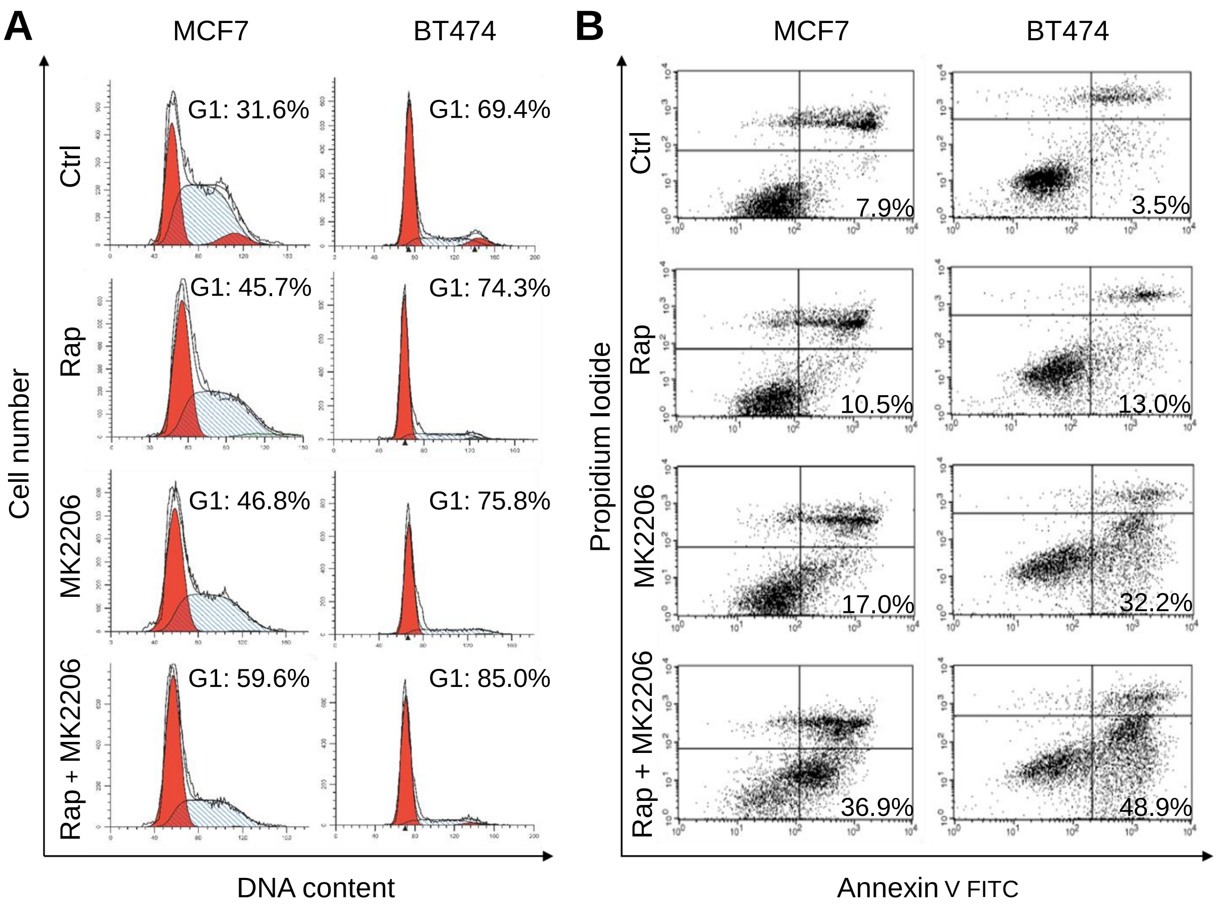

Supplementary Figure 1: Combination of rapamycin and MK2206 profoundly induces G1 arrest and apoptosis in MCF7 and BT474 cells. Cells were treated with 50 nM rapamycin (Rap) and 1 µM MK2206, alone or in combination or DMSO as control for 24 h (A) and 72 h (B), respectively. The fraction of cells in G1 (A) was determined by flow cytometry. Apoptosis in cells (B) was monitored by propidium iodide/Annexin V staining and flow cytometry.

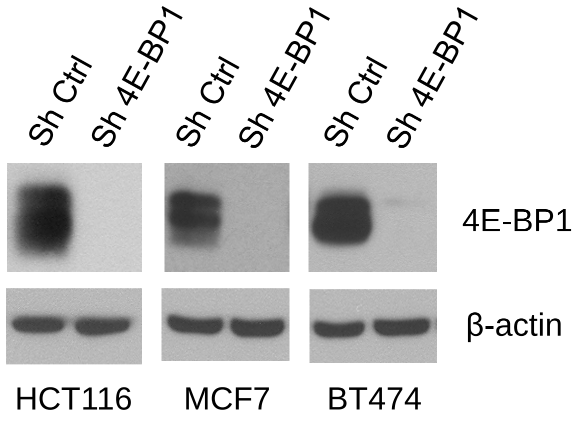

Supplementary Figure 2: Silencing 4E-BP1 expression by a shRNA. HCT116, MCF7 and BT474 cells with stable expression of Ctrl shRNA or 4E-BP1 shRNA were subjected to Western blot analysis for 4E-BP1. β-actin served as a loading control.

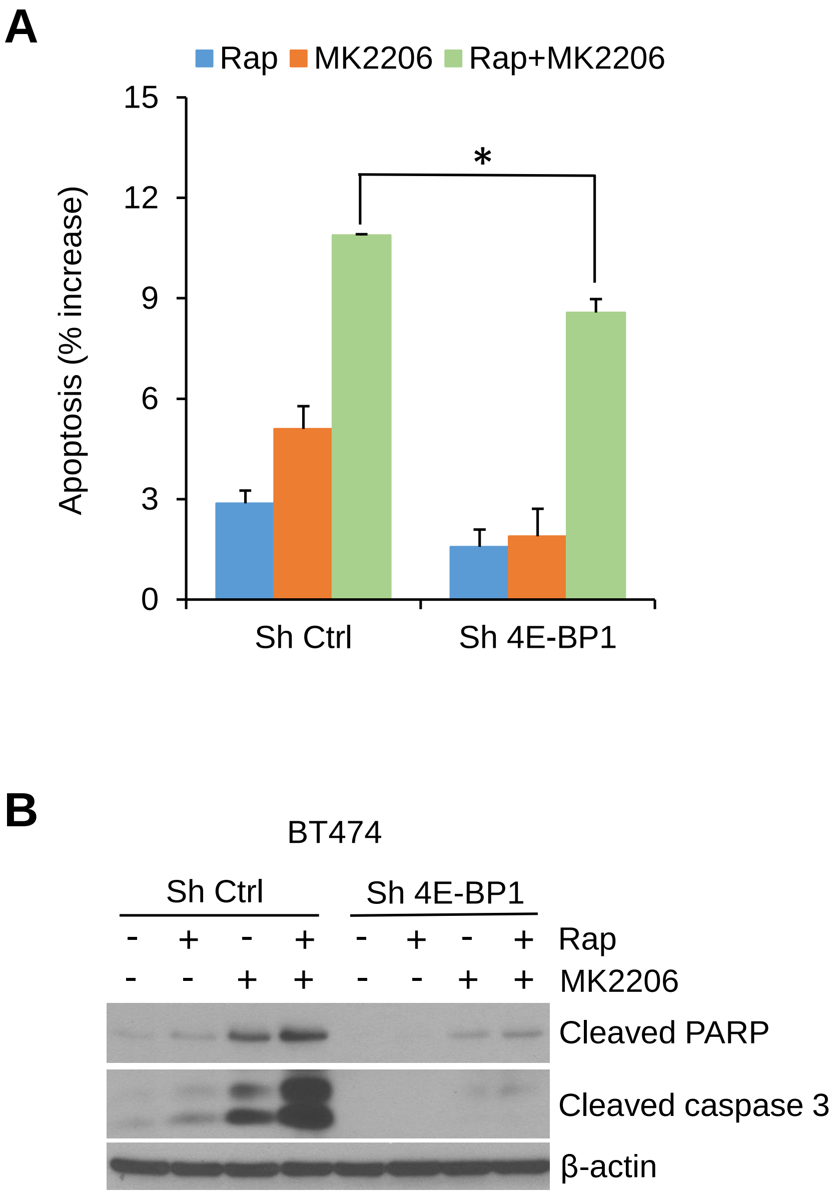

Supplementary Figure 3: Silencing 4E-BP1 expression in BT474 cells significantly reduces apoptosis induced by the combined inhibition of AKT and mTORC1. (A) BT474 cells with stable expression of control shRNA or 4E-BP1 shRNA were treated with 50 nM rapamycin (Rap) and 1 µM MK2206, alone or in combination or DMSO as control for 72 h followed by flow cytometry analysis for apoptosis. The results are expressed as the increased level of apoptosis by subtracting each of the DMSO-treated controls. $^{*}P < 0.03$ for combination of Rap and MK2206 in Sh 4E-BP1 cells versus that in Sh Ctrl cells. Data shown in the graph represent the mean ± S.E.M. (n=2). (B) BT474 cells with stable expression of control shRNA or 4E-BP1 shRNA were treated with 50 nM rapamycin (Rap) and 1 µM MK2206, alone or in combination or DMSO as control for 12 h followed by Western blot analysis for cleaved PARP and caspase-3. β-actin served as a loading control.

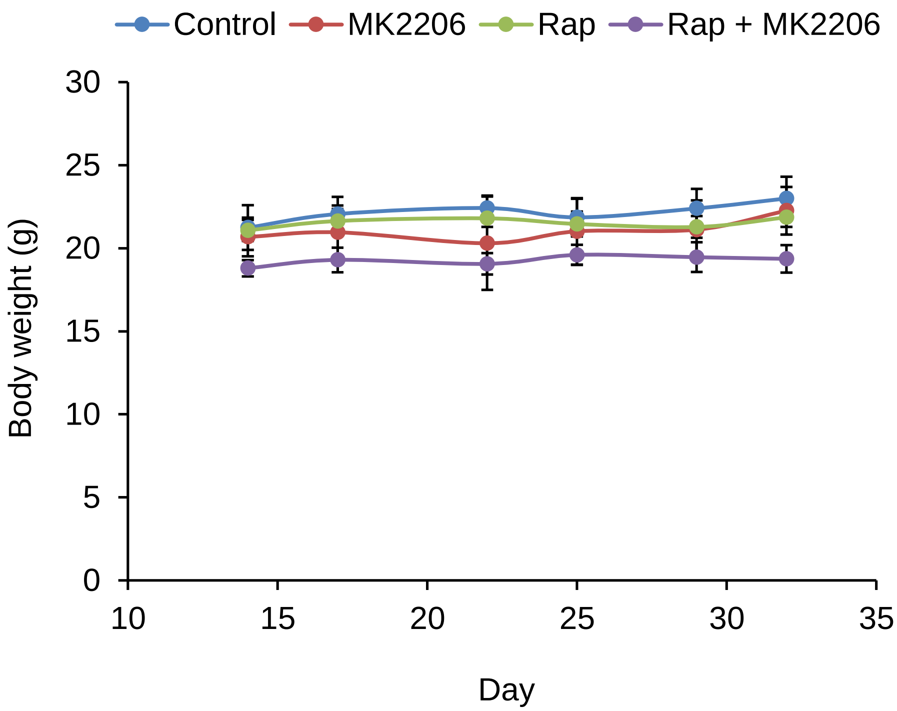

Supplementary Figure 4: Chronic treatment with rapamycin and MK2206, alone or in combination, does not cause weight loss in mice. Mice bearing MCF7 xenografts were treated with rapamycin (Rap) (4 mg/kg five times/week), MK2206 (100 mg/kg three times/week), combination of both drugs, or vehicle control. The mouse body weight was measured in control and treated groups using a weighing scale. The results represent the mean body weight ± S.E.M. (n = 5 mice per group).

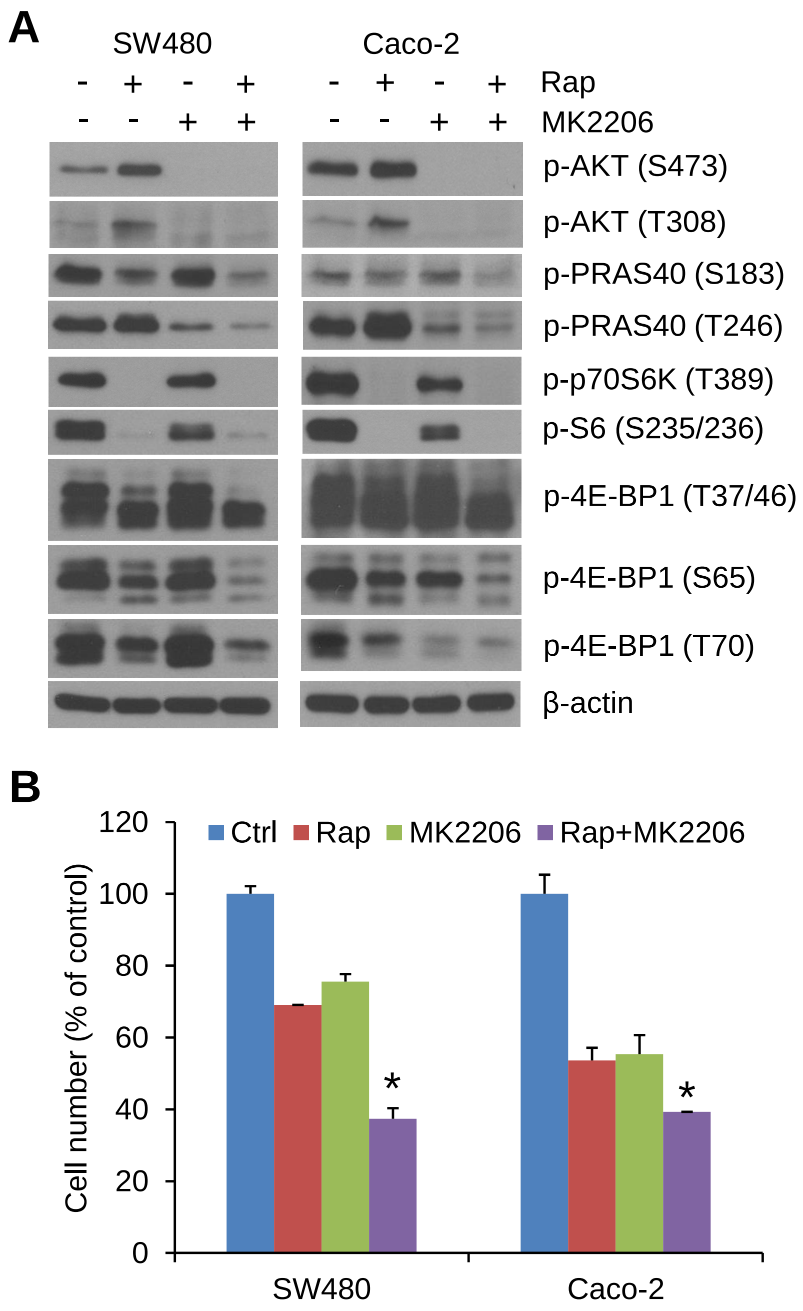

Supplementary Figure 5: Combination of MK2206 and rapamycin is required for effective inhibition of both PRAS40 and 4E-BP1 phosphorylation and cell growth in *KRAS* mutant SW480 and *KRAS/PIK3CA* wild-type Caco-2 colon cancer cells. (A) Cells were treated with 50 nM rapamycin (Rap) and 1 µM MK2206, alone or in combination or DMSO as control for 12 h followed by Western blot analysis for the indicated proteins. (B) The growth of the indicated cells was assessed after 3 days of treatment with 50 nM Rap and 1 µM MK2206, alone or in combination or DMSO as control (Ctrl). The results are shown as a percentage of cell number relative to DMSO-treated control cells. *$P < 0.03$ for combination of Rap and MK2206 versus DMSO Ctrl, Rap or MK2206.

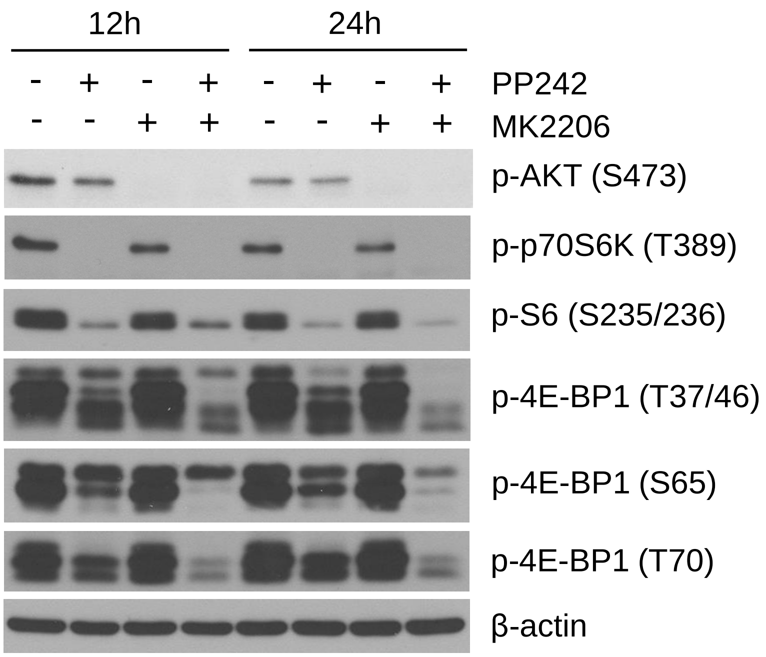

Supplementary Figure 6: MK2206 enhances the inhibitory effect of PP242 on 4E-BP1 phosphorylation in SW620 colon cancer cells. SW620 cells were treated with 1 µM PP242 and 1 µM MK2206, alone or in combination or DMSO as control for 12 h and 24 h followed by Western blot analysis for the indicated proteins.
